# Supplementary material for: Internet addiction and sleep quality among medical students during the COVID-19 pandemic: A multinational cross-sectional survey
Source: PLoS One. 2021 Nov 5;16(11):e0259594. doi: 10.1371/journal.pone.0259594 (PMC8570473; doi:10.1371/journal.pone.0259594)
Supplement: Attachment — Submitted filename: PONE-D-21-19421 internet.pdf [file pone.0259594.s002.pdf]

PLOS ONE

Internet addiction and sleep quality among medical students during the COVID-19 pandemic: A multinational cross-sectional survey

--Manuscript Draft--

**Manuscript Number:**	PONE-D-21-19421
**Article Type:**	Research Article
**Full Title:**	Internet addiction and sleep quality among medical students during the COVID-19 pandemic: A multinational cross-sectional survey
**Short Title:**	internet addiction and sleep quality among medical students during the COVID-19 pandemic
**Corresponding Author:**	Amir H. Pakpour, Ph.D.<br>Qazvin University of Medical Sciences<br>Qazvin, Qazvin IRAN, ISLAMIC REPUBLIC OF
**Keywords:**	COVID-19; Internet; addiction; sleep hygiene; medical students; Pandemic
**Abstract:**	Introduction<br><br>The emergence of the COVID-19 pandemic has affected the lives of many people, including medical students. The present study explored internet addiction and changes in sleep patterns among medical students during the pandemic and assessed the relationship between them.<br><br>Methods<br><br>A cross-sectional study was carried out in seven countries, the Dominican Republic, Egypt, Guyana, India, Mexico, Pakistan, and Sudan, using a convenience sampling technique, an online survey comprising demographic details, information regarding COVID-19, the Pittsburgh Sleep Quality Index (PSQI), and the Internet addiction test (IAT).<br><br>Results<br><br>In total, 2749 participants completed the questionnaire. Of the total, 67.6% scored above 30 in the IAT, suggesting the presence of an Internet addiction, and 73.5% scored equal and above 5 in the PSQI, suggesting poor sleep quality. Internet addiction was found to be significant predictors of poor sleep quality, causing 13.2% of the variance in poor sleep quality. Participants who reported COVID-19 related symptoms had disturbed sleep and higher internet addiction levels when compared with those who did not. Participants who reported a diagnosis of COVID-19 reported poor sleep quality. Those living with a COVID-19 diagnosed patient reported higher internet addiction and worse sleep quality compared with those who did not have any COVID-19 patients in their surroundings.<br><br>Conclusion<br><br>The results of this study suggest that internet addiction and poor sleep quality are two issues that require addressing amongst medical students. Medical training institutions should do their best to minimize their negative impact, particularly during the current COVID-19 pandemic.
**Order of Authors:**	Muhammad Junaid Tahir
	Najma Iqbal Malik
	Irfan Ullah
	Hamza Rafique Khan
	Shahida Perveen
	Rodrigo Ramalho

Powered by Editorial Manager® and ProduXion Manager® from Aries Systems Corporation

	Ahsun Rizwan Siddiqi
	Summaiya Waheed
	Mahmoud Mohamed Mohamed Shalaby
	Domenico De Berardis
	Samiksha Jain
	Gautham Lakshmipriya Vetrivendan
	Harshita Chatterjee
	William Xochitun Gopar Franco
	Muhammad Ahsan Shafiq
	Naira Taiba Fatima
	Maria Abeysekera
	Qudsia Sayyeda
	Shamat Fathi Shamat
	Wajeeha Aiman
	Qirat Akhtar
	Arooj Devi
	Anam Aftab
	Sheikh Shoib
	Chung-Ying Lin
	Amir H. Pakpour, Ph.D.
Opposed Reviewers:	
Additional Information:	
**Question**	**Response**
**Financial Disclosure**<br><br>Enter a financial disclosure statement that describes the sources of funding for the work included in this submission. Review the submission guidelines for detailed requirements. View published research articles from *PLOS ONE* for specific examples.<br><br>This statement is required for submission and **will appear in the published article** if the submission is accepted. Please make sure it is accurate.	The funders had no role in study design, data collection and analysis, decision to publish, or preparation of the manuscript.

**Unfunded studies**<br>Enter: *The author(s) received no specific funding for this work.*<br><br>**Funded studies**<br>Enter a statement with the following details:<br>• Initials of the authors who received each award<br>• Grant numbers awarded to each author<br>• The full name of each funder<br>• URL of each funder website<br>• Did the sponsors or funders play any role in the study design, data collection and analysis, decision to publish, or preparation of the manuscript?<br>• **NO** - Include this sentence at the end of your statement: *The funders had no role in study design, data collection and analysis, decision to publish, or preparation of the manuscript.*<br>• **YES** - Specify the role(s) played.<br><br>* typeset	
**Competing Interests**<br><br>Use the instructions below to enter a competing interest statement for this submission. On behalf of all authors, disclose any competing interests that could be perceived to bias this work—acknowledging all financial support and any other relevant financial or non-financial competing interests.<br><br>This statement is required for submission and **will appear in the published article** if the submission is accepted. Please make sure it is accurate and that any funding sources listed in your Funding Information later in the submission form are also declared in your Financial Disclosure statement.<br><br>View published research articles from *PLOS ONE* for specific examples.	The authors have declared that no competing interests exist.

Powered by Editorial Manager® and ProduXion Manager® from Aries Systems Corporation

**NO authors have competing interests**<br><br>Enter: *The authors have declared that no competing interests exist.*<br><br>**Authors with competing interests**<br><br>Enter competing interest details beginning with this statement:<br><br>*I have read the journal's policy and the authors of this manuscript have the following competing interests: [insert competing interests here]*<br><br>* typeset	
**Ethics Statement**<br><br>Enter an ethics statement for this submission. This statement is required if the study involved:<br><br>• Human participants<br>• Human specimens or tissue<br>• Vertebrate animals or cephalopods<br>• Vertebrate embryos or tissues<br>• Field research<br><br>Write "N/A" if the submission does not require an ethics statement.<br><br>General guidance is provided below. Consult the submission guidelines for detailed instructions. **Make sure that all information entered here is included in the Methods section of the manuscript.**	University of Sargodha, Pakistan

**Format for specific study types**<br><br>**Human Subject Research (involving human participants and/or tissue)**<br>• Give the name of the institutional review board or ethics committee that approved the study<br>• Include the approval number and/or a statement indicating approval of this research<br>• Indicate the form of consent obtained (written/oral) or the reason that consent was not obtained (e.g. the data were analyzed anonymously)<br><br>**Animal Research (involving vertebrate animals, embryos or tissues)**<br>• Provide the name of the Institutional Animal Care and Use Committee (IACUC) or other relevant ethics board that reviewed the study protocol, and indicate whether they approved this research or granted a formal waiver of ethical approval<br>• Include an approval number if one was obtained<br>• If the study involved *non-human primates*, add *additional details* about animal welfare and steps taken to ameliorate suffering<br>• If anesthesia, euthanasia, or any kind of animal sacrifice is part of the study, include briefly which substances and/or methods were applied<br><br>**Field Research**<br><br>Include the following details if this study involves the collection of plant, animal, or other materials from a natural setting:<br>• Field permit number<br>• Name of the institution or relevant body that granted permission	
**Data Availability**<br><br>Authors are required to make all data underlying the findings described fully available, without restriction, and from the time of publication. PLOS allows rare exceptions to address legal and ethical concerns. See the PLOS Data Policy and FAQ for detailed information.	Yes - all data are fully available without restriction

A Data Availability Statement describing where the data can be found is required at submission. Your answers to this question constitute the Data Availability Statement and **will be published in the article**, if accepted.<br><br>**Important:** Stating 'data available on request from the author' is not sufficient. If your data are only available upon request, select 'No' for the first question and explain your exceptional situation in the text box.<br><br>Do the authors confirm that all data underlying the findings described in their manuscript are fully available without restriction?	
**Describe where the data may be found in full sentences. If you are copying our sample text, replace any instances of XXX with the appropriate details.**<br><br>• If the data are **held or will be held in a public repository**, include URLs, accession numbers or DOIs. If this information will only be available after acceptance, indicate this by ticking the box below. For example: *All XXX files are available from the XXX database (accession number(s) XXX, XXX.).*<br>• If the data are all contained **within the manuscript and/or Supporting Information files**, enter the following: *All relevant data are within the manuscript and its Supporting Information files.*<br>• If neither of these applies but you are able to provide **details of access elsewhere**, with or without limitations, please do so. For example:<br><br>*Data cannot be shared publicly because of [XXX]. Data are available from the XXX Institutional Data Access / Ethics Committee (contact via XXX) for researchers who meet the criteria for access to confidential data.*<br><br>*The data underlying the results presented in the study are available from (include the name of the third party*	The data underlying the results presented in the study are available from the corresponding authors

and contact information or URL).<br>• This text is appropriate if the data are owned by a third party and authors do not have permission to share the data.<br><br>* typeset	
Additional data availability information:	

June 13, 2021

Dear Editor-in-Chief,

We submit our manuscript "***Internet addiction and sleep quality among medical students during the COVID-19 pandemic: A multinational cross-sectional survey***" to be considered for publication in *PLOS ONE.* This is a methodological study that has not been submitted to any other journal or published in full or in part in any form. The authors accept full responsibility for the accuracy of all content, including findings, citations, quotations, and references contained within the manuscript; release and assign all rights for the publication of the manuscript to **PLOS**; and declare no conflicts of interests related to the research or the manuscript. This manuscript has not been previously presented as an abstract, a presentation, or a manuscript. Authorship has been granted only to those individuals who have contributed substantially to the research or manuscript, and is agreed by all authors. This study was approved by the several institutional review boards, which we have detailed described in our manuscript. All authors declare no conflicts of interest.

We hereby certify 1) that all authors have made a substantial contribution to the information or material submitted for publication; 2) that all have read and approved the final manuscript; 3) that they have no direct or indirect commercial financial incentive associated with publishing the article; 4) that the source of extra-institutional funding is indicated; 5) that the manuscript or portions thereof are not under consideration by another journal or electronic publication and have not been previously published; 6) No listed authors on the manuscript have any relationship with Iranian government agency that has a primary function; 7) No listed authors are submitting this manuscript as an official representative or on behalf of the Iranian government.

Sincerely,

Corresponding authors
Amir H. Pakpour, PhD
Chung-Ying Lin, PhD

Manuscript
Click here to access/download;Manuscript;Internet addiction and sleep quality.docx

Internet addiction and sleep quality among medical students during the COVID-19 pandemic: A multinational cross-sectional survey

Muhammad Junaid Tahir[1,2], Najma Iqbal Malik[3], Irfan Ullah[4], Hamza Rafique Khan[5], Shahida Perveen[3], Rodrigo Ramalho[6], Ahsun Rizwan Siddiqi[7], Summaiya Waheed[8], Mahmoud Mohamed Mohamed Shalaby[9], Domenico De Berardis[10], Samiksha Jain[11], Gautham Lakshmipriya Vetrivendan[12], Harshita Chatterjee[13], William Xochitun GoparFranco[14], Muhammad Ahsan Shafiq[15], Naira Taiba Fatima[16], Maria Abeysekera[17], Qudsia Sayyeda[18], Shamat Fathi Shamat[19], Wajeeha Aiman[20], Qirat Akhtar[21], Arooj Devi[22], Anam Aftab[7], Sheikh Shoib[23], Chung-Ying Lin[24], Amir H Pakpour[25*]

[1]Ameer-ud-Din Medical College, Affiliated with University of Health and Sciences, Lahore, Pakistan

[2]Lahore General Hospital, Lahore, Pakistan

[3]University of Sargodha, Sargodha, Pakistan

[4]Kabir Medical College, Gandhara University, Peshawar, Pakistan

[5]Quaid-E-Azam Medical College, Bahawalpur, Pakistan

[6]Department of Social and Community Health, School of Population Health, The University of Auckland, Auckland, New Zealand

[7] Wah Medical College, affiliated with University of Health Sciences, Wah, Pakistan

[8] Dow Medical College, Dow University of Health Sciences, Karachi, Pakistan

[9]Faculty of Medicine, Ain Shams University, Cairo, 13763, Egypt

[10]NHS, Department of Mental Health, Psychiatric Service for Diagnosis and Treatment, Hospital "G. Mazzini", ASL 4, 64100 Teramo, Italy

[11]Guntur Medical College, Guntur, Andhra Pradesh, India

[12]Karpaga Vinayaga Institute of Medical Sciences and Research Centre, Maduranthagam, Tamilnadu, India

[13]Texila American University, Georgetown, Guyana

[14]University of Guadalajara, Puerto Vallarta, Mexico

[15]Holy Family Hospital, Rawalpindi, Pakistan

[16]Changsha Medical University, Changsha, Hunan, China

[17]Autonomous University of Santo Domingo

[18]Red Crescent Clinic Tampa Bay, Tampa, Florida, USA

[19]National Ribat University Medical School, Khartoum, Sudan

[20]Nishtar Medical College and Hospital, Multan, Pakistan

[21]Aziz Bhatti Shaheed Hospital, Gujrat, Punjab, Pakistan

[22]Shaheed Mohtarma Benazir Bhutto Medical University, Larkana, Pakistan

[23]Department of Psychiatry, Jawahar Lal Nehru Memorial Hospital, Srinagar, India

[24]Institute of Allied Health Sciences, National Cheng Kung University Hospital, College of Medicine, National Cheng Kung University, Tainan, Taiwan

[25]School of Health and Welfare, Jönköping University, Sweden

***Corresponding Author**

Dr. Amir H. Pakpour, PhD

Professor, Social Determinants of Health Research Center, Research Institute for Prevention of Non-Communicable Diseases, Qazvin University of Medical Sciences, ShahidBahounar BLV, Qazvin,3419759811, Iran.

Email: Pakpour_Amir@yahoo.com

Emails and ORCID IDs:

Muhammad Junaid **Tahir**: junaid262626@gmail.com, https://orcid.org/0000-0002-0335-6681

Najma Iqbal **Malik**: najmamalik@gmail.com, najma.iqbal@uos.edu.pk, https://orcid.org/0000-0002-3521-1014

Irfan **Ullah**: irfanullahecp2@gmail.com, https://orcid.org/0000-0003-1100-101X

Shahida **Perveen**:shahidaperveen11@gmail.com, https://orcid.org/0000-0002-0808-4099

Ahsun Rizwan **Siddiqi**: ahsun23@gmail.com, https://orcid.org/0000-0003-4699-6221

Summaiya **Waheed**: summaiyawaheed.97@gmail.com, https://orcid.org/0000-0003-1199-3039

Mahmoud Mohamed Mohamed **Shalaby**: shalabym1421998@gmail.com, https://orcid.org/0000-0001-7237-4616

Domenico De **Berardis**: domenico.deberardis@aslteramo.it , https://orcid.org/0000-0003-4415-5058

Samiksha **Jain**: Samiksha190404@gmail.com, https://orcid.org/0000-0001-9258-3159

Gautham Lakshmipriya **Vetrivendan**: gauthamvetri@gmail.com, https://orcid.org/0000-0003-0106-173X

Harshita **Chatterjee**: harshu.chatterjee02@gmail.com, https://orcid.org/0000-0002-7732-472X

William Xochitun Gopar **Franco**: willgofra@gmail.com, https://orcid.org/0000-0003-0668-4581

Muhammad Ahsan **Shafiq**: ahsanshafiq90@gmail.com, https://orcid.org/0000-0002-2255-4544

Naira Taiba **Fatima**: nairataibafatima89@gmail.com, https://orcid.org/0000-0002-2341-9694

Maria **Abeysekera**: abeysekeramaria@gmail.com, https://orcid.org/0000-0002-3222-5402

Qudsia **Sayyeda**: sayyeda.qudsia@gmail.com, https://orcid.org/0000-0001-7078-585X

Shamat Fathi **Shamat**: shamatfathi246@gmail.com, https://orcid.org/0000-0001-6746-5038

Wajeeha **Aiman**: wajeeha.dogar174@gmail.com, https://orcid.org/0000-0001-8306-8788

Qirat **Akhtar**: qiraatakhtar38@yahoo.com, https://orcid.org/0000-0001-7169-6365

Arooj **Devi**: aroojdevi@gmail.com, https://orcid.org/0000-0002-7156-6787

Anam **Aftab**: anaamaftab@gmail.com, https://orcid.org/0000-0003-3434-9529

Sheikh **Shoib**: Sheikhshoib22@gmail.com

Chung-Ying **Lin**: cylin36933@gmail.com, https://orcid.org/0000-0002-2129-4242

Amir H **Pakpour**: Pakpour_Amir@yahoo.com, https://orcid.org/0000-0002-8798-5345

Abstract

Background: The emergence of the COVID-19 pandemic has affected the lives of many people, including medical students. The present study explored internet addiction and changes in sleep patterns among medical students during the pandemic and assessed the relationship between them.

Methods: A cross-sectional study was carried out in seven countries, the Dominican Republic, Egypt, Guyana, India, Mexico, Pakistan, and Sudan, using a convenience sampling technique, an online survey comprising demographic details, information regarding COVID-19, the Pittsburgh Sleep Quality Index (PSQI), and the Internet addiction test (IAT).

Results: In total, 2749 participants completed the questionnaire. Of the total, 67.6% scored above 30 in the IAT, suggesting the presence of an Internet addiction, and 73.5% scored equal and above 5 in the PSQI, suggesting poor sleep quality. Internet addiction was found to be significant predictors of poor sleep quality, causing 13.2% of the variance in poor sleep quality. Participants who reported COVID-19 related symptoms had disturbed sleep and higher internet addiction levels when compared with those who did not. Participants who reported a diagnosis of COVID-19 reported poor sleep quality. Those living with a COVID-19 diagnosed patient reported higher internet addiction and worse sleep quality compared with those who did not have any COVID-19 patients in their surroundings.

Conclusion: The results of this study suggest that internet addiction and poor sleep quality are two issues that require addressing amongst medical students. Medical training institutions should do their best to minimize their negative impact, particularly during the current COVID-19 pandemic.

Keywords: Covid-19, internet, addiction, sleep hygiene, medical students, pandemic

Background

The Internet has completely revolutionized the world in the past few decades, with the 21st century witnessing explosive growth in worldwide Internet usage [1]. This global digitalization has provided better opportunities for education, communication, banking, businesses, health-seeking, and social interaction [1]. But, while the Internet can significantly optimize human life when used in moderation, its uncontrolled use can lead to maladaptive behaviors, including Internet addiction, also termed as problematic Internet use or pathological Internet use [2].

The World Health Organization has been addressing the public health implications of excessive use of the Internet since 2014 [3]. Internet addiction has been characterized as "excessive or poorly controlled preoccupations, urges or behaviors regarding computer use and Internet access that lead to impairment or distress" [4]. Internet addiction has also been linked to other psychiatric disorders, such as attention deficit and hyperactivity, as well as alcohol abuse [5]. Prolonged Internet usage adversely impacts both physical and mental health.

Sleep problems had a clear correlation with mental health as well as psychiatric illnesses. Specifically disorders related to anxiety and mood. Azad et al. [6] mentioned that poor sleep quality was not only related to various sleep related disorders but it can further cause exacerbate or develop the mental illness. In addition to this, Ford and Kamerow [7] mentioned that sleep disturbance may play role of a cause, a comorbidity, or a symptom of psychiatric disorder in future.

Cellini et al. [8] analyzed the changes in the sleep pattern with the use of electronic gadgets among the university students and workers. Their findings showed the participants spent

a great time on internet while in bed, slept late night and woke up late in the morning, all of which affected the quality of sleep and led to an increase in the levels of stress, anxiety and depression. This resulted in neglect of household chores, and a decline in the performance and productivity of the day. It was found that participants spent more time online than was needed and used to get excited and anticipate about when they will go online and some would get annoyed or yell if they are bothered when online.

Pathological Internet usage can also negatively affect the circadian rhythm causing insomnia and other sleep disturbances [9, 10, 11, 12]. Cellini et al. [8] found that in young adult workers and university students, increased digital media use before bedtime adversely affected sleep quality and resulted in increased levels of stress, anxiety, and depression. With the increasing popularity of smartphones, and all its advanced technology, the use of a smartphone before sleep has become a habit for students, one that could increase sleep latency and reduce sleep duration [13]. Moreover, it was found that teenagers who had trouble falling asleep or sustaining sleep were progressively inclined to have an Internet addiction, and people who were dependent on the Internet had their basic circadian rhythms altered [14]. Clear-cut impact that lockdown pose on sleep disorders is still less explained and understood, but it is evident that the students are spending time in bed as well as use of digital devices around bedtime also increased as compare to past, moreover, they are going to bed as well as got up from bed later, which is worse situation for their sleep quality [15].Both these issues are of concern. On the one hand, insufficient sleep and poor sleep quality are closely related to poor memory and weakened learning abilities, which hurt the academic performance of students and can also result in other growth and developmental disorders [16, 17, 18]. On the other hand, among students, excessive

Internet usage can lead to grey matter atrophy, which negatively affects one's ability to concentrate and hinders their decision-making capacity [19].

The COVID-19 pandemic has led to an inevitable surge in the use of digital technologies due to the physical distancing norms and nationwide lockdowns [20]. People and organizations all over the world have had to adjust to new ways of work and life [20], and medical schools are no exception. Medical schools in both developed and developing countries have utilized modern technologies to bring effective changes in medical education [21]. Over the years, but particularly during COVID-19, medical students have faced significant changes in their education, often finding themselves attending online classes and studying independently [22]. Medical students have shifted to virtual learning, forcing students to spend more time online [23]. Also, due to the decrease in face to face social interactions and the increased time spent indoors during COVID-19, there is a growing dependence on social media and online entertainment platforms for social interaction [24] that resulted in heavily indulgence on internet and consequently affected physical and mental health in daily life of students. Additionally, the demographic characteristics under focus of present study included Age gender residence, family system, country, family size, type of medical university, year of MBBS, smoking history, health status, COVID 19 Related symptoms, Been diagnosed with COVID-19 by a health profession, Have you been living with a person diagnosed with COVID-19 diagnosis, Are you following COVID-19 Standard Operating Procedures. Some of these have empirical support like age [25] gender [25, 6, 15], family system [15], year of MBBS [15], smoking history [25] and physical health status [6, 25] were previously explored while other few demographic were exploratory findings of present study.

Empirically it was evident that excessive internet usage may significantly affect the sleep cycle of the person which leads to insomnia, irregular sleep patterns and excessive daytime sleepiness [12, 26]. Current study evaluated the effect of COVID-19 pandemic on the use of internet and subsequently, sleeping habits among the medical students. It also viewed at how the sleeping habits were altered by the excessive or problematic use of internet. To date, relatively few studies have examined the effect of the current COVID-19 pandemic on Internet addiction and sleep quality particularly among medical students. University graduates from all around the world reported a common problem i.e. sleep deprivation. Azad et al. [6] compared these sleep related issues among medical students and economics and law undergraduates. He mentioned that medical students rank the highest on the prevalence regarding poor sleep and even worse quality of life in comparison of students from other subject groups. Matter of fact that segregate these medical graduates from other peer groups included their overall lifestyle, attitude towards study and more academic pressure. Palatty et al. [27] further differentiated economics and Law graduates had more stressful academic demands and responsibilities in their study programs in comparison to students belong to various other academic discipline. This fact explained the reason of poor sleep among medical students compared to other student population.

It was mentioned in previous studies that COVID-19 harmed performance and had worse effect on sleep patterns, as due to lockdown, this Blanco et al. [15] moreover, these studies also compare groups on basis of demographics, so it was also followed in this study. However, few demographics were similar in previous studies while others are specific to Pakistani culture and hence are exploratory analysis on basis of demographic groups in present study. The present study aims to fill this gap. With this regard, several hypotheses were made below: (i) internet

addiction is positively associated with poor sleep quality; (ii) subdomains in the internet addiction are significant predictors for poor sleep quality; (iii) students with issues related to COVID-19 have high levels of internet addiction and poor sleep quality; and (iv) students with different demographics have different levels of internet addiction and sleep quality.

Methods

Study participants

A survey research design was applied, using a non-probability convenience sampling technique. The research team developed a consent form and a questionnaire using shared Google ® forms. Data collection took place between June and July 2020 after study approval from the University of Sargodha, Pakistan. The target population was students aged 15 to 44 years old in medical colleges from the Dominican Republic, Egypt, Guyana, India, Mexico, Pakistan, and Sudan. First, the relevant institutions' concerned authorities provided their informed consent, authorizing the study to be conducted with students who attended their institutions. Then, potential participants were contacted directly by the researcher through their active email addresses and WhatsApp numbers provided by their institute co-coordinators. A web link to the questionnaire was then shared with them, and informed consent was obtained before participating in the study. The bulk of the data collection was from countries which delivered their curriculum in English and they could understand English language easily. Hence, responses from these places were collected in an English version of questionnaire. Translation to Spanish was done for collecting data from only two countries i.e. Dominican Republic and Mexico, in order to reduce the language barrier in their countries. For these two countries, a single questionnaire (file attached) containing both languages were circulated, and participants could

read the question in English or Spanish according to their ease. Participants were not given any monetary benefits for participating in the study. To restrict duplication of responses, respondents were asked to provide email address, as only one response can be submitted through one email address. After data collection, prior to data analysis, data was reviewed and cleaned incomplete responses were discarded and only complete responses per subject were analyzed further for hypotheses testing. The Ethic Committee of University of Sargodha was approved the study procedure (SU/PSY/786-S, April 09,2020). Consent was informed by all participants. All participants provided online informed consent**.**

Measure

The questionnaire bank included demographic questions and two scales, the Pittsburgh sleep quality index (PSQI) and the Internet addiction Test (IAT) [28, 29]. The consent form and the questionnaire were developed in two languages, English and Spanish.

Demographics information

Collected demographics information included age, gender, country of residence, whether they lived in an urban or rural area, family system (joint or nuclear family), whether the participant attended a private or a public medical university, and the participants' year of study and smoking history; plus, the following COVID-19 related questions: (i) do you have any COVID-19 related symptoms? (ii) have you been diagnosed with COVID-19 by a health professional? (iii) have you been living with a person diagnosed with COVID-19? (iv) are you following COVID-19 Standard Operating Procedures (SOPs)?

<u>Pittsburgh Sleep Quality Index (PSQI)</u>

PSQI is a self-rated questionnaire [28] that assesses sleep quality and disturbances over a 1-month time interval through 19 individual items, four open-ended questions, and 14 questions rated on a Likert scale from 0-3 with 3 reflecting the extreme negative. These 19 items generate seven component scores, i.e., subjective sleep quality, sleep latency, sleep duration, habitual sleep efficiency, sleep disturbances, use of sleeping medication, and daytime dysfunction, and the sum of scores for these seven components yields a PSQI global score of sleep quality which ranges from 0 to 21. A total sum of 5 or more indicates "poor" quality of sleep. The scale has acceptable reliability, Cronbach's α of 0.914 [30] and 0.73 [31]. The Reliability of the PSQI in present was excellent (Cronbach's α of 0.91). Raniti et al. [31] validated the single factor structure of PSQI in an adolescent sample and mentioned covariation among poor sleep efficiency, latency and poor duration within this age students which is considerable.

Internet addiction test (IAT)

The IAT is a 20-item questionnaire that measures characteristics and behaviors associated with compulsive use of the Internet [29]. Each item is weighted along a Likert-scale continuum that ranges from 0 = less extreme behavior to 5 = most extreme behavior. Total scores that range from 0 to 30 points are read as representing a normal level of Internet usage, a score range of 31 to 49 indicates the presence of a mild level of Internet addiction, scores of 50 to 79 indicate a moderate level, and scores of 80 to 100 indicate a severe level of Internet addiction. IAT has high Cronbach's α of 0.93 [32]. The Reliability Coefficient of the IAT was excellent in present study (Cronbach's α = 0.77). Significant correlations of subscales confirm the internal consistency of the scales used in this study. Further, this scale consisted of 5 subscales, whose reliability was also calculated. They were salience (Cronbach's α = 0.77), excessive use

(Cronbach's α = 0.77), neglect work (Cronbach's α = 0.77), anticipation (Cronbach's α = 0.77), lack of control (Cronbach's α = 0.77), neglect social life. (Cronbach's α = 0.77).

Statistical Analysis

All collected data were scored and analyzed. Descriptive statistics including mean, standard deviation (SD), range, skewness, alpha reliability coefficient of all scales and their subscales were computed. Further, mean, standard deviation, range, and skewness were computed for the PSQI and IAT, including their total score and subscale scores. The mean differences in PSQI and IAT (including total and subscale scores) between demographic variables were explored and compared using independent t-tests and effect size (i.e., Cohen's *d*). Analyses of variance (ANOVAs) were carried out to examine the PSQI and IAT differences (including total and subscale scores) between different sizes of family. Descriptive statistics using mean scores in the PSQI and IAT total and subscale scores to compare country differences were calculated as well. Pearson correlations were used to examine the zero-order bivariate correlations between studied variables. Finally, multiple regression models were constructed to examine the association between sleep and internet addiction. The first step is to examine whether the internet addiction is associated with the overall sleep (i.e., using the total score of PSQI). After identifying significant association between overall sleep and internet addiction, subscale scores of the PSQI were used to examine the association between different domains of sleep and internet addiction. For all the regression models, total and subscale scores of the PSQI were the dependent variables in the regression models; the subscale scores of all the IAT were the independent variables; demographic variables (including age [adolescent vs. adult], gender [male vs. female] residence [rural vs. urban], family system [joint vs. nuclear], medical university sector [public vs. private], year of MBBS [junior vs. senior], smoking history [yes vs.

no], health status [poor vs. good], COVID-19 related symptoms [yes vs. no], COVID-19 diagnosis [yes vs. no], live with COVID-19 infected individuals [yes vs. no], and COVID-19 standard operating procedures [yes vs. no]) were controlled variables.

Results

The final sample size was comprised of 2749 participants, 991 (36%) male and 1758 (64%) female participants. Further demographic information is presented in Table 1. A majority of the participants were from Pakistan (n=1009; 36.7%) and India (n=939; 34.2%). Most participants, 2311(84.1%), resided in urban areas, and 1936 (70.4%) belonged to a nuclear family system. Also, most participants studied at a public university, 1678 (61%), and 2035 (74%) were juniors (1st year to 4th year) and 714 (26%) seniors (5th and 6th year). Regarding the COVID-19 related questions, most participants, 2398 (87.2%) reported following COVID-19 SOPs. Similarly, most participants, 2621 (95.5%), reported no COVID-19 related symptoms, and only 189 (6.9%) had been diagnosed with COVID-19 by a health professional.

[Insert Table 1 here]

Overall, 67.6% (n = 1859) of the sample scored above 30 in the IAT, indicating the presence of an Internet addiction. Among the 1859 participants; 40.0% (n = 1099) scored between 31 and 49 in the IAT, indicating the presence of a mild level Internet addiction; 25.5% (n = 700) scored between 50 and 79, indicating a moderate level Internet addiction; and 2.2% (n= 60) scored 80 and over, indicating a severe dependence. Additionally, 73.5% (n = 2007) of the total sample had a global score of 5 or more on the PSQI, indicating poor sleep quality. Detailed scores in PSQI and IAT are presented in Table 2.

[Insert Table 2 near here]

Table 3 reports comparisons of the mean differences and effect sizes in IAT and PSQI total scores between the collected demographic data. Significant differences were observed in almost all the comparisons, except for the IAT scores between COVID-19 diagnosis and following COVID-19 standard operating procedures or not; the PSQI score between following COVID-19 standard operating procedures or not. Table 4 shows the comparisons of PSQI and IAT scores (including total and subscale scores) between different sizes of family. These are exploratory findings as results showed that subjective sleep quality and sleep duration were the best among participants from large families; the use of sleep medication was also the highest in large families. Scores on Internet addiction, including the total score, the salience, neglect of work, and neglect social life subscale scores were the highest in large families. Table 5 further presents the comparisons of PSQI and IAT scores (including total and subscale scores) across countries using descriptive statistics. More specifically, the highest mean score for IAT and PSQI was reported in participants from Pakistan (IAT=100, PSQI=19), followed by India (IAT=97, PSQI=16) and Egypt (IAT=81, PSQI=17); the lowest score was reported in participants from the Dominican Republic (IAT=26.47, PSQI=5.81).

[Insert Tables 3 to 5 near here]

Table 6 presents the inter-correlations between PSQI and IAT scores (including total score and subscale scores). Except for sleep efficiency subscale in the PSQI, all the PSQI scores and IAT scores were positively and significantly associated. Given the significance of correlations were supported, the multiple regression models assessing the associations between sleep and Internet addiction are conducted. The multiple regression models showed excessive internet use and neglect in social life, as in lockdown both were high and affected negatively on

sleep quality were the most important factors significantly explaining poor sleep quality in the present sample (Table 7).

[Insert Tables 6 and 7 near here]

Discussion

The present study explored the impact of COVID-19 on the lifestyle of medical college students across seven different countries. In particular, it explored the relationship between the presence or absence of symptoms or a diagnosis of COVID, as well as the use of SOPs, on Internet addiction and sleep quality. It also explored the relationship between Internet addiction and sleep quality and other variables, such as place of residence, overall health, and smoking history. Finally, it examined the relationship between Internet addiction as an independent variable and sleep quality as a dependent variable. To compare cutoff with previous cutoff scores, percentile ranks were calculated and results identified no major differences in score range. Superficially, minimum value of range was slightly shifted from 40 to 51 in the total score of IAT. However, the cutoff score is within the range of 40-69.

Participants who reported COVID-19 symptoms reported disturbed sleep and scored higher on Internet addiction than those who did not report any symptoms. Also, participants who reported a diagnosis of COVID-19 made by a health professional reported poor sleep quality, whereas results on Internet addiction were found non-significant. Similarly, participants who were living with someone with a COVID-19 diagnosis also scored higher on Internet addiction and reported worse sleep quality when compared to those who were not. The study found a non-

significant effect of compliance with COVID-19 preventive SOPs on Internet addiction and sleep quality.

As previously reported by Garcia-Priego [33], public health emergencies can cause, trigger, or worsen mental health concerns, plus, they can also be related to a high prevalence of low to mild levels of Internet addiction. Regarding COVID-19, a study by Siste et. al. [34] found that mental health issues and sleep disruptions were related to Internet addiction, and it was especially prevalent in groups with proximity to COVID-19. Fear of COVID-19 contraction and rampant misinformation about COVID-19 could have contributed to these results.

In the present study, adolescents and male participants scored higher on Internet addiction when compared to adults and female participants respectively. However, both male and female were non significantly different in terms of their sleep quality. Similar results were reported by Kim et al. [35]. Similar results were found in other studies too [6, 25,15]. where individuals with Internet addiction tended to be males of young age and found no significant gender difference in sleep quality. The authors further reported that adults with Internet addiction reported high difficulty in initiating and maintaining sleep, had a non-restorative sleep cycle, showed daytime functional impairment, and their duration of sleep was more than 10 hours on weekdays.

The present study found no significant effect of the place of residence on Internet addiction. However, it did have a significant effect on sleep quality, as participants living in urban areas had poorer sleep quality when compared to those living in rural areas. Participants living in joint families and those attending a private medical university scored higher on Internet addiction and poorer sleep quality when compared to those living in nuclear families and attending a public medical university, respectively. Some opposite results were reported by Jahan

et al. [36], who found that living with their family was associated with better sleep quality. In the present study, juniors scored higher on Internet addiction than seniors, while senior students reported poorer sleep quality. Here, a similar result was previously reported by Cheng et al. [37], and Blanco et al. [15], who found that undergraduates (although not separated in junior or senior years) had poorer sleep quality than postgraduates.

Participants with a smoking history and self-reported poor health scored higher on Internet addiction and also reported poor sleep quality when compared to those who did not smoke and reported good health. Previous studies found similar results in this regard. For example, Jahan et al. [36] also found that non-smokers had a better sleep quality. A study conducted in Taiwan found that students who smoked had altered sleeping patterns and were less likely to have good sleep quality. Liu et al. [38] and Cheng et al. [37] also reported that people who reported poor sleep quality also smoked excessive and indulged in high Internet surfing.

Additionally, this study also indicated significant mean differences on all scales and subscales other than sleep latency, sleep efficiency, and daytime dysfunction. Results showed that sleep quality was best in students from large families, better in medium-sized families, and poor in small families. Further, Internet addiction was highest in large families, moderate in small families, and was least in middle families.

Results suggest that the IAT and all its subscales have a significant positive correlation with PSQI, and all its subscales, except sleep efficiency, with which it had a non-significant relationship. In summary, Internet addiction was a significant predictor of poor sleep quality. Previous studies have also found similar results. Jahan et al. [36] study with medical college students of Bangladesh also found as Internet addiction increased, the level of poor sleep quality also increased. The study by Canan et. al. [12], conducted with Turkish high school students,

also reported an association between Internet addiction and impaired sleep. Similarly, the study by Tsitsika [39], conducted in seven European countries, found that the prevalence of sleep problems was higher among students with an Internet-addiction. Finally, a systematic review conducted by Lam [10], found that Internet gaming addiction and problematic Internet use were related to sleep problems, including insomnia and poor sleep quality, although more so the latter than the former.

The Coronavirus has caused an extraordinary crisis in many fields; therefore, many countries are struggling to get out of the crisis. Mulyadi et al. [40] examine the relationship between sleep duration and anxiety and internet usage duration. Their results showed a significant relationship between sleep duration and anxiety; and internet usage duration with anxiety. Another study from the Middle East region (Kuwait, Saudi Arabia) reported by Alheneidi et al. [41] showed an association between loneliness and Problematic Internet Use (PIU), and an association between loneliness and the number of hours spent online. Those who reported greater loneliness also obtained frequent news about the pandemic from social media. Their ANOVA analyses further showed a dose-response between the predictors and PIU. Moreover, Lin [42] mentioned that the coronavirus disease 2019 (COVID-19) outbreak has significantly disrupted normal activities globally. During this epidemic, people around the world were expected to encounter several mental health challenges. Internet addiction may become a serious issue among teenagers. The prevalence of internet addiction was found to be 24.4% during this period [42]. Additionally, previous studies report an increase in gaming addiction and internet use with detrimental impact on psychosocial well-being. Fernandes et al. [43] compared the impact of lockdown on internet use among adolescents via their habits between before and during the pandemic. They found the relationship between gaming addiction, internet use and

COVID-19 worries and showed that adolescents generally have increased their use of social media sites and streaming services. Further, those who had a high level of gaming addiction, compulsive internet use and social media use also reported high scores of depression, loneliness, escapism, poor sleep quality and anxiety related to the pandemic. Their findings indicated that, regardless of country of residence, the COVID-19 outbreak has had a significant effect on adolescent internet use and psychosocial well-being.

The present study also found the varied effect of different constructs of Internet addiction on different constructs of sleep quality. For example, anticipation, a subscale of the IAT, was a significant predictor of sleep disturbance, and lack of control was a significant predictor of the use of sleep medication. Neglecting social life, a domain of the IAT, was a significant predictor of sleep disturbance, use of sleep medication, and subjective sleep quality. Work neglect, a sub-construct of IAT, was a significant predictor of sleep disturbance, sleep duration, and daytime dysfunction. Lastly, the salience of Internet addiction and excessive use of the Internet (subscales of IAT) were significant predictors of sleep disturbance, use of sleep medication, subjective sleep quality, sleep duration, daytime dysfunction, and sleep latency. A previous study conducted by Lin et al. [25] also reported that Internet addiction was significantly associated with subjective sleep disturbance, use of sleep medication, sleep quality, sleep duration, daytime dysfunction, and sleep latency.

The strength of this study is that it has focused on the area of research where not much work has been done during the COVID-19 pandemic. Other strengths include the large sample size, use of an international sample (increasing generalizability), and the potentially important implications for mental health and sleep interventions among medical students. However, our study had several limitations as follows: Firstly, the convenience sampling method was used and

due to voluntary participation, there was a possibility of selection bias. Secondly, because this was a cross sectional study, we were unable to establish causal inferences. Thirdly, most participants belonged to Pakistan and India due to which it is difficult to generalize the results. Finally, the varying prevalence of COVID-19 in different countries accompanied with the subsequent imposition of standard operating procedures (SOPs) and the degree of lockdown individually, could have been a contributor to bias and was not taken into account.

Looking at the results, future studies should focus on how to allow the medical students to use internet in a balanced manner so as to prevent the development of internet addiction during such lockdown conditions and, devise measures to improve the quality of sleep among medical students.

Conclusion

The present study suggest the possibility that the COVID-19 pandemic, along with its public health measures, has had a significant impact on Internet use and sleep quality amongst medical students. The study found that about 67.6% of the total sample scored over 30 in the IAT, indicating the possibility of Internet addiction. Also, 73.5% of the total sample scored above 5 in the PSQI, indicating poor sleep quality. The presence of COVID-19 related symptoms was associated with disturbed sleep and higher scores in the IAT, and a diagnosis of COVID-19 was associated with poor sleep quality. Similarly, living with someone with a COVID-19 diagnosis was associated with a higher score on the IAT and worse sleep quality. These findings suggest the importance of providing medical students with coping strategies that would prevent pathological Internet usage and poor sleep quality. Further, psychological mindedness was

impacted due to COVID-19 because student did not expected and trained to handle this situation. This study highlight the need to design some sort of training to deal with such pandemic situation, which was previously not given to student sample. During the COVID-19 pandemic, educational institutions around the globe are recommended to provide adequate psychological support, to minimize the adverse effects of the pandemic.

Funding: None.

Competing interests: The authors have declared that no competing interests exist.

References

1. Chaudhari B, Menon P, Saldanha D, Tewari A, Bhattacharya L. Internet addiction and its determinants among medical students. Ind Psychiatry J. 2015;24(2):158-162. doi:10.4103/0972-6748.181729

2. Kuss DJ, Lopez-Fernandez O. Internet addiction and problematic Internet use: A systematic review of clinical research. World J Psychiatry. 2016;6(1):143-176. Published 2016 Mar 22. doi:10.5498/wjp.v6.i1.143

3. World Health Organization. Public health implications of excessive use of the Internet and other communication and gaming platforms. Available from https://www.who.int/news-room/detail/13-09-2018-public-health-implications-of-excessive-use-of-the-internet-and-other-communication-and-gaming-platforms. Accessed Sep 3, 2020.

4. Shaw M, Black DW. Internet addiction: definition, assessment, epidemiology and clinical management. CNS Drugs. 2008;22(5):353-365. doi:10.2165/00023210-200822050-00001

5. Ho RC, Zhang MW, Tsang TY, et al. The association between internet addiction and psychiatric co-morbidity: a meta-analysis. BMC Psychiatry. 2014;14:183. Published 2014 Jun 20. doi:10.1186/1471-244X-14-183

6. Azad MC, Fraser K, Rumana N, et al. Sleep disturbances among medical students: a global perspective. J Clin Sleep Med. 2015;11(1):69-74. Published 2015 Jan 15. doi:10.5664/jcsm.4370

7. Ford DE, Kamerow DB. Epidemiologic study of sleep disturbances and psychiatric disorders. An opportunity for prevention?. JAMA. 1989;262(11):1479-1484. doi:10.1001/jama.262.11.1479

8. Cellini N, Canale N, Mioni G, Costa S. Changes in sleep pattern, sense of time and digital media use during COVID-19 lockdown in Italy. J Sleep Res. 2020;29(4):e13074. doi:10.1111/jsr.13074

9. Alimoradi Z, Lin CY, Broström A, et al. Internet addiction and sleep problems: A systematic review and meta-analysis. Sleep Med Rev. 2019;47:51-61. doi:10.1016/j.smrv.2019.06.004

10. Lam LT. Internet gaming addiction, problematic use of the internet, and sleep problems: a systematic review. Curr Psychiatry Rep. 2014;16(4):444. doi:10.1007/s11920-014-0444-1

11. Jenaro C, Flores N, Gómez-Vela M, González-Gil F, Caballo C. Problematic internet and cell-phone use: Psychological, behavioral, and health correlates. Addiction research & theory. 2007;15(3):309-20.doi:10.1080/16066350701350247

12. Canan F, Yildirim O, Sinani G, Ozturk O, Ustunel TY, Ataoglu A. Internet addiction and sleep disturbance symptoms among T urkish high school students. Sleep and Biological Rhythms. 2013;11(3):210-3. https://doi.org/10.1111/sbr.12022

13. Yang SY, Chen KL, Lin PH, Wang PY. Relationships among health-related behaviors, smartphone dependence, and sleep duration in female junior college students. Social Health and Behavior. 2019;2(1):26. doi: 10.4103/SHB.SHB_44_18

14. Zhang MWB, Tran BX, Huong LT, et al. Internet addiction and sleep quality among Vietnamese youths. Asian J Psychiatr. 2017;28:15-20. doi:10.1016/j.ajp.2017.03.025

15. Romero-Blanco C, Rodríguez-Almagro J, Onieva-Zafra MD, Parra-Fernández ML, Prado-Laguna MDC, Hernández-Martínez A. Sleep Pattern Changes in Nursing Students during the COVID-19 Lockdown. Int J Environ Res Public Health. 2020;17(14):5222. Published 2020 Jul 20. doi:10.3390/ijerph17145222

16. Curcio G, Ferrara M, De Gennaro L. Sleep loss, learning capacity and academic performance. Sleep Med Rev. 2006;10(5):323-337. doi:10.1016/j.smrv.2005.11.001

17. Kwon JA, Lee M, Yoo KB, Park EC. Does the duration and time of sleep increase the risk of allergic rhinitis? Results of the 6-year nationwide Korea youth risk behavior web-based survey. PLoS One. 2013;8(8):e72507. Published 2013 Aug 27. doi:10.1371/journal.pone.0072507

18. Kim JH, Park EC, Lee SG, Yoo KB. Associations between time in bed and suicidal thoughts, plans and attempts in Korean adolescents. BMJ Open. 2015;5(9):e008766. Published 2015 Sep 4. doi:10.1136/bmjopen-2015-008766

19. Yuan K, Qin W, Liu Y, Tian J. Internet addiction: Neuroimaging findings. Commun Integr Biol. 2011;4(6):637-639. doi:10.4161/cib.1787120. De' R, Pandey N, Pal A. Impact of digital surge during Covid-19 pandemic: A viewpoint on research and practice. Int J Inf Manage. 2020;55:102171. doi:10.1016/j.ijinfomgt.2020.102171

21. Haque M, Rahman NA, Majumder MA, et al. Internet use and addiction among medical students of Universiti Sultan Zainal Abidin, Malaysia. Psychol Res Behav Manag. 2016;9:297-307. Published 2016 Nov 14. doi:10.2147/PRBM.S119275

22. Verma A, Verma S, Garg P, Godara R. Online Teaching During COVID-19: Perception of Medical Undergraduate Students [published online ahead of print, 2020 Jun 27]. Indian J Surg. 2020;1-2. doi:10.1007/s12262-020-02487-2

23. Meo SA, Abukhalaf AA, Alomar AA, Sattar K, Klonoff DC. COVID-19 Pandemic: Impact of Quarantine on Medical Students' Mental Wellbeing and Learning Behaviors. Pak J Med Sci. 2020;36(COVID19-S4):S43-S48. doi:10.12669/pjms.36.COVID19-S4.2809

24. Dixit A, Marthoenis M, Arafat SMY, Sharma P, Kar SK. Binge watching behavior during COVID 19 pandemic: A cross-sectional, cross-national online survey. Psychiatry Res. 2020;289:113089. doi:10.1016/j.psychres.2020.113089

25. Lin PH, Lee YC, Chen KL, Hsieh PL, Yang SY, Lin YL. The Relationship Between Sleep Quality and Internet Addiction Among Female College Students. Front Neurosci. 2019;13:599. Published 2019 Jun 12. doi:10.3389/fnins.2019.00599

26. Van den Bulck J. Television viewing, computer game playing, and Internet use and self-reported time to bed and time out of bed in secondary-school children. Sleep. 2004;27(1):101-104. doi:10.1093/sleep/27.1.101

27. Palatty PL, Fernandes E, Suresh S, Baliga M. Comparison of sleep pattern between medical ald law students. Sleep and Hypnosis. 2011;13(1-2):15-8.

28. Zhang C, Zhang H, Zhao M, et al. Reliability, Validity, and Factor Structure of Pittsburgh Sleep Quality Index in Community-Based Centenarians. Front Psychiatry. 2020;11:573530. Published 2020 Aug 31. doi:10.3389/fpsyt.2020.573530

29. Young KS. Caught in the net: How to recognize the signs of internet addiction--and a winning strategy for recovery. John Wiley & Sons; 1998 Feb 27.

30. Samaha AA, Fawaz M, El Yahfoufi N, et al. Assessing the Psychometric Properties of the Internet Addiction Test (IAT) Among Lebanese College Students. Front Public Health. 2018;6:365. Published 2018 Dec 17. doi:10.3389/fpubh.2018.00365

31. Raniti MB, Waloszek JM, Schwartz O, Allen NB, Trinder J. Factor structure and psychometric properties of the Pittsburgh Sleep Quality Index in community-based adolescents. Sleep. 2018;41(6):zsy066.. https://doi.org/10.1093/sleep/zsy066

32. Lai CM, Mak KK, Watanabe H, Ang RP, Pang JS, Ho RC. Psychometric properties of the internet addiction test in Chinese adolescents. Journal of Pediatric Psychology. 2013;38(7):794-807. https://doi.org/10.1093/jpepsy/jst022

33. Garcia-Priego BA, Triana-Romero A, Pinto-Galvez SM, Duran-Ramos C, Salas-Nolasco O, Reyes MM, de la Medina AR, Troche JM. Anxiety, depression, attitudes, and internet addiction during the initial phase of the 2019 coronavirus disease (COVID-19) epidemic: A cross-sectional study in Mexico. MedRxiv. 2020 Jan 1. doi: https://doi.org/10.1101/2020.05.10.20095844

34. Siste K, Hanafi E, Sen LT, et al. The Impact of Physical Distancing and Associated Factors Towards Internet Addiction Among Adults in Indonesia During COVID-19 Pandemic: A Nationwide Web-Based Study. Front Psychiatry. 2020;11:580977. Published 2020 Sep 3. doi:10.3389/fpsyt.2020.580977

35. Kim K, Lee H, Hong JP, et al. Poor sleep quality and suicide attempt among adults with internet addiction: A nationwide community sample of Korea. PLoS One. 2017;12(4):e0174619. Published 2017 Apr 6. doi:10.1371/journal.pone.0174619

36. Jahan SM, Hossain SR, Sayeed UB, Wahab A, Rahman T, Hossain A. Association between internet addiction and sleep quality among students: a cross-sectional study in Bangladesh. Sleep and Biological Rhythms. 2019;17(3):323-9.

37. Cheng SH, Shih CC, Lee IH, et al. A study on the sleep quality of incoming university students. Psychiatry Res. 2012;197(3):270-274. doi:10.1016/j.psychres.2011.08.011

38. Liu JT, Lee IH, Wang CH, Chen KC, Lee CI, Yang YK. Cigarette smoking might impair memory and sleep quality. J Formos Med Assoc. 2013;112(5):287-290. doi:10.1016/j.jfma.2011.12.006

39. Tsitsika A, Janikian M, Schoenmakers TM, et al. Internet addictive behavior in adolescence: a cross-sectional study in seven European countries. Cyberpsychol Behav Soc Netw. 2014;17(8):528-535. doi:10.1089/cyber.2013.0382

40. Mulyadi S, Prabowo H, Salve HR, Ayuningsih AM. Sleep Duration, Internet Use Duration and Anxiety on University Student during Covid-19 Pandemic. http://www.ieomsociety.org/detroit2020/papers/466.pdf

41. Alheneidi H, AlSumait L, AlSumait D, Smith AP. Loneliness and Problematic Internet Use during COVID-19 Lock-Down. Behav Sci (Basel). 2021;11(1):5. Published 2021 Jan 6. doi:10.3390/bs11010005

42. Lin MP. Prevalence of Internet Addiction during the COVID-19 Outbreak and Its Risk Factors among Junior High School Students in Taiwan. Int J Environ Res Public Health. 2020;17(22):8547. Published 2020 Nov 18. doi:10.3390/ijerph17228547

43. Fernandes B, Biswas UN, Mansukhani RT, Casarín AV, Essau CA. The impact of COVID-19 lockdown on internet use and escapism in adolescents. Revista de Psicología Clínica con Niños y Adolescentes. 2020;7(3):59-65.

Table 1. ***Demographic characteristics of the sample (N=2749)***

	Demographic variable	n	%
	Age		
1.	Adolescent (15-20)	1065	38.7
2.	Adult (21-44)	1684	61.3
	Gender		
1.	Male	991	36
2.	Female	1758	64
	Country		
1.	Dominican republic	138	5
2.	Egypt	133	4.8
3.	Guyana	178	6.5
4.	India	939	34.2
5.	Mexico	218	7.9
6.	Pakistan	1009	36.7
7.	Sudan	134	4.9
	Residence		
1.	Rural	438	15.9
2.	Urban	2311	84.1
	Family system		
1.	Joint family	812	29.6
2.	Nuclear family	1936	70.4
	Family size (no of family members)		
1.	Small family (1 to 5)	1775	64.6
2.	Medium family (6 to 10)	863	31.4
3.	Large family (11 to 50)	111	4.0
	Medical university sector		
1.	Public	1678	61
2.	Private	1071	39
	Year of MBBS		
1.	Juniors (1st year to 4th year)	2035	74

2.	Seniors (5th and 6th year, graduates and internee)	714	26
	Any smoking history		
1.	No	2477	90.1
2.	Yes	272	9.9
	Health status		
	1.Poor health	1048	38.1
	2. Good health	1701	61.9
	COVID-19 related symptoms		
1.	No	2621	95.5
2.	Yes	125	4.5
	Been diagnosed with COVID-19 by a health profession		
1.	No	2690	93.1
2.	Yes	189	6.9
	Have you been living with a person diagnosed with COVID-19 diagnosis		
1.	No	2560	93.1
2.	Yes	189	6.9
	Are you following COVID-19 Standard Operating Procedures		
1.	No	351	12.8
2.	Yes	2398	87.2

Table 2. ***Descriptive information for the Pittsburgh Sleep Quality Index (PSQI), Internet Addiction Test (IAT), and their subscales (N = 2749)***

					Range		
Scales	Subscales	*M*	*SD*	AC	Potential	Actual	Skewness
Global PSQI	Total	6.73	3.16	.77	0-21	.00-19	.43
Component 1	Subjective sleep quality	1.09	.81	-	0-3	0-3	.46
Component 2	Sleep latency	.80	.88	-	0-3	0-3	.17
Component 3	Sleep duration	.80	.88	-	0-3	0-3	.17
Component 4	Sleep efficiency	1.05	1.12	-	0-3	0-3	.90
Component 5	Sleep disturbance	1.14	.54	-	0-3	0-5	.82
Component 6	Use of sleep medication	.19	.55	-	0-3	0-3	.29
Component 7	Daytime dysfunction	1.07	.82	-	0-3	0-3	.36
IAT	Total	39.65	17.84	.91	0-5	0-100	.31
	Salience	9.10	5.16	.75	0-5	0-25	.42
	Excessive use	10.82	4.96	.71	0-5	0-25	.25
	Neglect work	5.84	3.64	.73	0-5	0-15	.38
	Anticipation	3.68	2.32	.43	0-5	0-10	.48
	Lack of control	7.16	3.63	.71	0-5	0-15	.15
	Neglect social life	3.02	2.08	.41	0-5	0-10	.64

Note. SQ = sleep quality; Dis = distress; CS = coping scale; AC = adaptive coping; MC = maladaptive coping; EI = emotional intelligence.

Table 3. ***Comparison of demographics on IAT and PSQI (N = 2749)***

		IAT		95% *CI*		*Cohen's d*	PSQI		95% *CI*		*Cohen's d*
Variables	*n*	*M*	*SD*	*UL*	*LL*		*M*	*SD*	*UL*	*LL*	
**Age**											
Adolescent (15-20)	1065	40.46	17.78	-.18	-.04	.07	6.47	3.14	-.18	-.67	.13
Adult (21-44)	1684	39.14	17.86				6.89	3.18			
**Gender**											
Male	991	40.98	17.48	3.47	.69	.11	6.61	3.15	.06	.69	.05
Female	1758	38.90	17.99				6.79	3.17			
**Residence**											
Rural	438	38.10	18.79	-.01	-3.65	.10	6.21	3.45	-.26	-.96	.18
Urban	2311	39.94	17.64				6.82	3.10			
**Family system**											
Joint family	812	41.19	18.62	3.65	.73	.12	6.99	3.36	.64	.11	.11
Nuclear family	1937	39.00	17.46				6.62	3.07			

**Medical university sector**											
Public	1678	38.19	17.85	-2.38	-5.11	.21	6.52	3.13	-.29	-.77	.17
Private	1071	41.94	17.58				7.06	3.19			
**Year of MBBS**											
Juniors	2035	40.66	17.83	5.40	2.37	.21	6.65	3.19	-.01	-.55	.09
Seniors	714	36.77	17.56				6.94	3.07			
**Smoking history**											
Yes	272	42.62	16.86	5.52	1.06	.18	7.61	3.37	1.37	.58	.30
No	2477	39.32	17.91				6.63	3.13			
**Health status**											
Poor health	1048	44.24	17.08	8.76	6.07	.42	7.63	3.17	1.69	1.21	.47
Good health	1701	36.82	17.71				6.17	3.03			
**COVID-19 related symptoms**											
Yes	125	42.94	17.34	6.64	.24	.19	8.08	3.33	1.97	.84	.43
No	2624	39.49	17.85				6.66	3.14			

**COVID-19 diagnosis**											
Yes	59	41.77	15.74	6.33	-1.98	.12	7.94	3.63	2.06	.42	.36
No	2690	39.60	17.88				6.70	3.15			
**Live with COVID-19 infected individual**											
Yes	189	43.59	16.14	6.64	1.81	.24	8.04	3.16	1.87	.94	.44
No	2560	39.36	17.92				6.63	3.14			
**Follow COVID-19 SOP**											
Yes	398	39.46	17.69	.53	-3.45	.08	6.70	3.12	.16	-.60	.06
No	351	40.92	18.78				6.92	3.48			

Note: IAT = Internet Addiction Test; PSQI = Pittsburgh Sleep Quality Index; SOP = Standard Operating Procedures

Table 4. ***Differences of studied variables in the present study between different sizes of family (N = 2749)***

	Small family (*n*=1775)		Medium family (*n* = 863)		Large family (*n* = 111)				
Variables	*M*	*SD*	*M*	*SD*	*M*	*SD*	*F*	η^2	Post-hoc comparison
Global PSQI	6.58	.08	6.96	3.28	7.32	3.45	6.23**	.11	1<2< 3
Total IAT	38.94	7.31	1.03	18.56	40.17	18.56	4.00**	.22	2<1<3
Subjective sleep quality	1.06	.78	1.14	.84	1.23	.87	4.19**	.05	1< 2<3
Sleep latency	1.35	1.00	1.40	1.00	1.33	.95	7.53	.02	-
Sleep duration	.76	84	.87	.92	1.01	1.00	7.67***	.05	1< 2<3
Sleep efficiency	1.06	1.12	1.04	1.11	.90	1.11	1.07	.008	-
Sleep disturbance	1.12	.54	1.17	.55	1.27	.57	5.40**	.03	2< 3<1
Use of sleep medication	.16	.49	.22	.62	.41	.75	12.01***	.06	1< 2<3
Daytime dysfunction	1.04	.80	1.11	.86	1.17	.84	2.45	.04	-
IAT-salience	8.85	5.03	9.51	5.35	9.87	5.61	5.97**	.14	1< 2<3
IAT excessive use	10.66	4.83	11.22	5.14	10.30	5.39	4.29**	.12	3< 1<2
IAT neglect work	5.80	3.55	5.91	3.78	6.01	3.87	.43	.03	1<2<3
IAT anticipation	3.59	2.26	3.86	2.41	3.77	2.47	3.98**	.08	1<3<2
IAT lack of control	7.07	3.53	7.36	3.77	6.99	4.15	1.99	.07	3< 1<2
IAT neglect social life	2.95	2.04	3.14	2.12	3.20	2.18	12.47*	.06	1<2<3

**p*< .05. ***p*< .01. ****p<.001*

Table 5. ***Differences of studied variables in the present study between countries (N = 2749)***

Variables	Pakistan (n=1009)	India (n=939)	Egypt (n=133)	Sudan (n=134)	Guyana (n=178)	Mexico (n=218)	Dominican Republic (n=138)
	M (SD)	*M (SD)*	*M (SD)*	*M (SD)*	*M (SD)*	*M (SD)*	*M (SD)*
Global PSQI	7.17 (3.16)	5.83 (2.97)	7.92 (3.58)	6.81 (3.64)	8.79 (2.40)	6.64 (2.81)	5.82 (2.88)
PSQI SSQ	1.14 (0.82)	0.89 (0.75)	1.44 (0.92)	1.18 (0.92)	1.53 (0.71)	1.18 (0.67)	1.0 (0.80)
PSQI SL	1.47 (1.00)	1.23 (1.03)	1.62 (1.04)	1.17 (1.07)	1.71 (0.66)	1.31 (0.95)	1.26 (0.94)
PSQI S.DUR	0.92 (0.92)	0.69 (0.78)	0.80 (1.01)	0.93 (1.04)	0.74 (0.81)	0.76 (0.88)	0.78 (0.89)
PSQI SE	1.07 (1.11)	0.96 (1.13)	1.17 (1.14)	1.15 (1.16)	1.23 (1.12)	1.15 (1.14)	0.91 (1.09)
PSQI S.DIST	1.17 (0.58)	1.03 (0.47)	1.26 (0.59)	1.21 (0.60)	1.63 (0.50)	1.10 (0.48)	1.11 (0.46)
PSQI UoSM	0.25 (0.64)	0.11 (0.42)	0.17 (0.53)	0.19 (0.56)	0.31 (0.54)	0.16 (0.52)	0.22 (0.64)
PSQI D.DYS	1.15 (0.82)	0.93 (0.80)	1.43 (0.88)	1.05 (0.87)	1.65 (0.67)	0.99 (0.76)	0.53 (0.65)
Total IAT	42.34 (18.58)	37.42 (17.61)	46.77 (16.32)	41.31 (16.45)	49.54 (11.77)	31.72 (13.25)	26.48 (14.21)
IAT S	10.12 (5.29)	8.32 (4.93)	11.18 (4.77)	9.65 (5.04)	12.39 (3.61)	5.72 (3.64)	5.57 (4.19)
IAT EU	11.51 (5.27)	9.95 (4.99)	12.41 (4.94)	11.31 (4.77)	12.35 (2.96)	10.38 (3.91)	8.49 (4.23)
IAT NW	6.59 (3.93)	6.19 (3.85)	6.56 (3.69)	4.93 (4.18)	7.23 (2.54)	3.66 (3.19)	2.39 (2.61)
IAT ANT	3.93 (2.36)	3.28 (2.21)	4.76 (2.39)	4.28 (2.54)	5.06 (1.68)	2.80 (2.04)	2.72 (1.98)
IAT LOC	7.45 (3.66)	7.12 (3.73)	8.36 (3.59)	7.51 (3.78)	7.93 (2.29)	6.11 (3.27)	4.58 (3.31)
IAT NSL	3.08 (2.17)	2.68 (1.98)	3.37 (2.13)	3.43 (2.39)	4.60 (1.64)	2.72 (1.69)	2.64 (1.69)

Note. PSQI = Pittsburgh Sleep Quality Index; IAT = Internet Addiction Test; SSQ = subjective sleep quality; SL =sleep latency; S.DUR = sleep duration; SE = sleep efficiency; S.DIST = sleep disturbance; UoSM =use of sleep medicine; D.DYS = daytime

dysfunction; S = salience; EU = excessive use; NW = neglect work; ANT = anticipation; LOC = lack of control; NSL = neglect social life.

Table 6. ***Correlation matrix for all the studied variables in the study (N = 2749)***

	Variables	1	2	3	4	5	6	7	8	9	10	11	12	13	14	15
1	Sum PSQI	-														
2	sum IAT	.36**	-													
3	Subjective sleep Quality	.68**	.28**	-												
4	Sleep Latency	.63**	.24**	.39**	-											
5	Sleep duration	.52**	.13**	.30**	.17**	-										
6	Sleep Efficiency	.44**	.01	.06**	.09**	.09**	--									
7	Sleep disturbance	.53**	.28**	.33**	.25**	.15**	.04*	-								
8	Use of Sleep Medicine	.39**	.14**	.18**	.13**	.10**	.02	.28**	-							
9	Daytime dysfunction	.61**	.37**	.42**	.26**	.13**	.05**	.35**	.21**	-						
10	IAT salience	.31**	.87**	.22**	.21**	.10**	.003	.27**	.15**	.30**	-					
11	IAT excessive use	.37**	.89**	.30**	.27**	.15**	.02	.23**	.12**	.35**	.71**	-				
12	IAT neglect work	.26**	.80**	.20**	.16**	.06**	.001	.21**	.09**	.34**	.60**	.65**	-			
13	IAT anticipation	.24**	.71**	.18**	.15**	.09**	.006	.22**	.11**	.25**	.60**	54**	53**	-		
14	IAT locus of control	.27**	.82**	.21**	.19**	.08**	.03	.18**	.05**	.30**	.62**	73**	65**	50**	-	
15	IAT neglect social life	.23**	.60**	.18**	.12**	.09**	.005	.21**	.16**	.20**	.52**	46**	39**	40**	36**	-

Note. 1 = Global PSQI; 2 = sum of internet addiction test (IAT); 3 = subjective sleep quality (SSQ); 4=sleep latency (SL); 5 = sleep duration (S.DUR); 6 = sleep efficiency (SE); 7 = sleep disturbance (S. DIST.); 8 = use of sleep medicine (UoSM); 9 = daytime

dysfunction (D.DYS); 10 = salience (S); 11 = excessive use (EU); 12 = neglect work (NW); 13 = anticipation (ANT); 14 = lack of control (LOC); 15 = neglect social life (NSL)

*$p < .05$. **$p < .01$.

Table 7. ***Multiple linear regression model for predicting sleep quality (N = 2749)***

	Dependent variables in sleep															
	Global PSQI		SSQ		SL		S.DUR		S.E		S.DIST		UoSM		D.DYS	
IV	B	SE	B	SE	B	SE	B	SE	B	SE	B	SE	B	SE	B	SE
Sal.	0.033	.017	-0.004	.005	0.010	.006	-0.003	.005	0.003	0.007	0.013***	0.003	0.011**	0.003	0.006	0.005
EU	0.168***	0.019	0.046***	0.005	0.052***	.006	0.033***	.006	0.003	0.008	0.003	0.003	0.006	0.004	0.026***	0.005
NW	-0.015	0.020	-0.006	0.005	-0.018**	.007	-0.009	.006	-0.006	0.008	0.005	0.004	-0.002	0.004	0.024***	0.005
Anti.	0.04	.031	0.006	0.008	0.004	.010	0.012	.009	-0.013	0.012	0.016**	0.006	0.005	0.006	0.010	0.008
LoC	-0.016	.024	0.006	0.006	-0.003	.008	-0.012	.007	0.013	0.009	-0.004	0.004	-0.015**	0.004	0.009	0.006
NSL	0.104***	.032	0.022**	0.008	0.004	0.011	0.010	0.01	0.010	0.013	0.023***	0.006	0.026***	0.006	0.006	0.008
R^2 (adj. R^2)	0.194 (0.189)		0.134 (0.128)		0.098 (0.092)		0.041 (0.034)		0.016 (0.009)		0.124 (0.119)		0.072 (0.065)		0.178 (0.173)	

Note. IV = independent variable; PSQI = Pittsburgh Sleep Quality Index; SSQ = subjective sleep quality; SL =sleep latency; S.DUR = sleep duration; SE = sleep efficiency; S.DIST = sleep disturbance; UoSM =use of sleep medicine; D.DYS = daytime dysfunction.

All the models controlled the demographic variables, including age (adolescent *vs.* adult), gender (male *vs.* female) residence (rural *vs.* urban), family system (joint *vs.* nuclear), medical university sector (public *vs.* private), year of MBBS (junior *vs.* senior), smoking history (yes *vs.* no), health status (poor *vs.* good), COVID-19 related symptoms (yes *vs.* no), COVID-19 diagnosis (yes *vs.* no), live with COVID-19 infected individuals (yes *vs.* no), and COVID-19 standard operating procedures (yes *vs.* no).

$^*p < .05$. $^{**}p < .01$. $^{***}p < .001$.

Other

Click here to access/download
Supporting Information
ETHICS.jpg

Other

Click here to access/download
Supporting Information
Project 1 Final. 13 August.sav
