# Supplementary material for: Internet addiction and sleep quality among medical students during the COVID-19 pandemic: A multinational cross-sectional survey
Source: PLoS One. 2021 Nov 5;16(11):e0259594. doi: 10.1371/journal.pone.0259594 (PMC8570473; doi:10.1371/journal.pone.0259594)
Supplement: Attachment — Submitted filename: 211019Response letter.doc [file pone.0259594.s004.doc]

October 19, 2021

Dear Dr. Mortazavi,

Thanks for giving us the opportunity to revise our work “**Internet addiction and sleep quality among medical students during the COVID-19 pandemic: A multinational cross-sectional survey (Manuscript ID PONE-D-21-19421R1)**”. After revising the manuscript, we have resubmitted it to be considered for publication on the *PLOS ONE.* We have systematically addressed the reviewers’ concerns point-by-point, and the revisions are presented in red font in the manuscript. We hope that the paper is now acceptable for publication in the *PLOS ONE*.

We look forward to your reply. Thank you for considering our manuscript.

Sincerely,

Corresponding Authors

**Responses to Editor:**

**Background:**

**1. Thank you for revising the manuscript and submitting it to PLOS ONE. A few things remain to be fixed but it has improved by the changes already made. We invite you to submit a revised version of the manuscript that addresses the points below:**

*Response:* Thank you for appreciating our previous revisions and thank you again for providing us additional constructive comments below to improve our work.

**2. The first sentence of the discussion section: “The present study explored the impact of COVID-19 on the lifestyle of medical college students across seven different countries.”**

**I do not agree with this statement. PLS kindly explain how this study explored the impact of the covid-19 on students’ lifestyle.**

**Please kindly discuss limitations of the study and potential sources of bias or imprecision. Discuss both direction and magnitude of any potential bias.**

**Please discuss the generalizability of the study results.**

*Response:* We have now revised the first sentence of the Discussion to precisely reflect what the present study investigates. That is, we have removed the claim of “impact” in the sentence as we did not have pre- and post-pandemic information to understand “impact”.

“The present study explored some aspects of the lifestyle (i.e., internet addiction and sleep) among medical college students across seven different countries during the period of COVID-19 pandemic.”

**3. Please kindly discuss limitations of the study and potential sources of bias or imprecision. Discuss both direction and magnitude of any potential bias.**

**Please discuss the generalizability of the study results.**

*Response:* Limitations of the study, including the potential sources of bias/imprecision, and the generalizability of the study results have been added.

“However, our study had several limitations as follows: Firstly, the convenience sampling method was used and due to voluntary participation, there was a possibility of selection bias. Specifically, those who did not have internet addiction or sleep problems were more likely than those who had such a problem to agree to participate. Therefore, the results of the present study may underestimate the prevalence of internet addiction and sleep problems. Secondly, because this was a cross sectional study, we were unable to establish causal inferences. That is, it is unclear whether internet addiction results in sleep problems, or the other way around. Future studies are thus needed to use a longitudinal design or case-control design to provide causal relationship evidence. Thirdly, most participants belonged to Pakistan and India due to which it is difficult to generalize the results. Specifically, the present study’s results may be prone more to Pakistani and Indians instead of other countries’ participants. Fourthly, the varying prevalence of COVID-19 in different countries accompanied with the subsequent imposition of SOPs and the degree of lockdown individually, could have been a contributor to bias and was not taken into account. Finally, the data collection was via the online mode; therefore, those who did not frequently surf on their internet during the COVID-19 pandemic might not be aware of this study. This may restrict the generalizability of the present findings. That is, those who were at very low level of internet addiction or internet use might not participate in the present study.”
